# A NanoLuc Luciferase Reporter Pseudorabies Virus for Live Imaging and Quantification of Viral Infection

**DOI:** 10.3389/fvets.2020.566446

**Published:** 2020-09-22

**Authors:** Yalin Wang, Hongxia Wu, Bing Wang, Hansong Qi, Zhao Jin, Hua-Ji Qiu, Yuan Sun

**Affiliations:** ^1^State Key Laboratory of Veterinary Biotechnology, Harbin Veterinary Research Institute, Chinese Academy of Agricultural Sciences, Harbin, China; ^2^College of Life Science and Agriculture Forestry, Qiqihar University, Qiqihar, China

**Keywords:** pseudorabies virus, NanoLuc luciferase, *in vivo*, image, mouse

## Abstract

Pseudorabies (PR), also known as Aujeszky's disease, is an acute infectious disease of pigs, resulting in significant economic losses to the pig industry in many countries. Since 2011, PR outbreaks have occurred in many Bartha-K61-vaccinated pig farms in China. The emerging pseudorabies virus (PRV) variants possess higher pathogenicity in pigs and mice than the strains isolated before. Here, a recombinant PRV (rPRVTJ-NLuc) stably expressing the NanoLuc (NLuc) luciferase fusion with the red fluorescent protein (DsRed) was constructed to trace viral replication and spread in mice. Moreover, both DsRed and NLuc luciferases were stably expressed in the infected cells, and there was no significant difference between wild-type and recombinant viruses in both growth kinetics and pathogenicity. Seven-week-old BALB/c mice were infected with 10^3^ 50% tissue culture infective dose rPRVTJ-NLuc and subjected to daily imaging. The mice infected with rPRVTJ-NLuc displayed robust bioluminescence that started 4 days postinfection (dpi), bioluminescence signal increased over time, peaked at 5 dpi, remained detectable for at least 6 dpi, and disappeared at 7 dpi, meanwhile, the increased flux accompanied by the spread of the virus from the injection site to the superior respiratory tract. However, the signal was also observed in the spinal cord, trigeminal ganglion, and partial region of the brain from separated tissues, not in living mice. Our results depicted a new approach to rapidly access the replication and pathogenicity of emerging PRVs in mice.

## Introduction

Pseudorabies (PR), a devastating disease in the pig industry worldwide, is characterized by neurological signs, respiratory signs, high morbidity, and mortality of piglets, whereas older pigs mostly exhibit respiratory and reproductive diseases (1). Pseudorabies virus (PRV) is the causative agent of the acute infectious PR in swine. Due to control efforts and strict implementation of national eradication programs, PR has been eradicated from domestic pigs in North America and several European countries. However, the disease is sporadic in many other countries, including China (2). In late 2011, there was a PR outbreak on many large pig farms where piglets were vaccinated with Bartha-K61vaccine, and quickly the disease occurred in six provinces in China. The mortality rate of infected piglets was from 10 to 50%, which caused great economic loss (3). Moreover, about 57.8% of 5,033 serum samples isolated during the year 2013 to 2016 were positive for PRV gE antibody, so PRV variant strains were still prevalent in China (4). The PRV variants shared 97.1–99.9% nucleotide (nt) and 96.6–99.5% amino acid (aa) homology with PRV reference strains, and they belonged to different clades (3). Mutation of glycoproteins C and D of PRV variants led to the escape from Bartha-K61 vaccine-induced immunity (5), so gE/gI/TK-deleted PRV variant strains as a substitute for Bartha-K61 vaccine were used in China to control PR at present. Except for the immunogenicity change, the pathogenicity of PRV variant strains enhanced compared with the classic strain in both pigs and mice; however, it is not clear about the mechanism of enhanced pathogenicity to its hosts (6).

Fluorescent proteins or luciferase-tagged viruses have been widely used in the researches of viral infection and replication mechanisms (7–9). Reporters that have luminescence properties possess greater advantages in some areas of virological research due to higher signal–noise ratio and sensitivity compared with fluorescent proteins (10). Many studies have demonstrated several significant advantages of investigating viral pathogenesis with imaging. Bioluminescence imaging (BLI) is a powerful alternative, enabling rapid measurements of viral load and tissue distribution (11–14). Spatial and temporal progression of infection can be quantified, and viral replication and dissemination in the animals can be identified (15). The traditional approaches for viral pathogenicity studies require the killing of animals at diverse time points for the determination of viral titers in excised organs and tissues, whereas BLI does not (16). In addition, BLI can identify unexpected sites or patterns of viral infection that could be missed if organs are not collected or if entire organs are not analyzed for viral titers (14). This approach has been exploited in multiple viruses, including dengue virus, herpes simplex virus type 1, Sindbis virus, influenza virus, and Sendai virus (12, 14, 15, 17, 18).

Here, we generated a recombinant PRV (rPRVTJ-NLuc) stably expressing the engineered luciferase variant NanoLuc and red fluorescent protein DsRed. NLuc is a 19-kDa luciferase engineered from the deep-sea shrimp that possesses ~150-fold greater specific activity than firefly luciferase (19, 20). The reporter gene of NLuc that fused with DsRed was inserted immediately downstream of the *US9* gene. The replication dynamics of the recombinant PRV is similar to PRVTJ. Moreover, rPRVTJ-NLuc possesses pathogenicity and lethality indistinguishable from those of PRVTJ in mice. These results demonstrated that the recombinant PRV was not attenuated both *in vitro* and *in vivo*. Furthermore, we reported the visualization of PRV infection in mice. These data suggest that imaging of the recombinant PRV can be used to rapidly assess the replication and pathogenicity characteristics of emerging PRVs; these will provide a reference for control PR caused by PRV variants.

## Materials and Methods

### Cells, Viruses, and Plasmids

The PRVTJ strain (GenBank accession number: KJ789182.1) was isolated from a pig farm outbreak in Tianjin of China, propagated in PK-15 cells, and stored at −70°C. PK-15 and Vero cells were maintained at 37°C with 5% carbon dioxide (CO_2_) in Dulbecco's modified Eagle medium (DMEM, Thermo-Fisher Scientific, Carlsbad, CA, United States) supplemented with 10% fetal bovine serum (Gibco, Grand Island, NY, United States). Both cell lines were obtained from the China Center for Type Culture Collection (Wuhan, China).

The left and right homologous arms (flanking the PRV *US9* gene, named as L and R) of transfer vector were amplified by using primer pairs P1S/P1R and P2S/P2R. The NLuc gene was amplified with primers P4S/P4R from pNL2.1 vector (Promega, Madison, WI) and inserted into the DsRed expressing vector pDsRed2-C1 (Clontech, USA) through *Eco*RI site firstly, and then, the inserted fragment together with CMV promoter and polyA terminator was amplified by PCR with primers P3S/P3R. The resulting L arm, R arm, and exogenous gene were amplified; 100–300 ng of DNA template was used per reaction for overlap PCR; the long DNA segment was cloned into blunt T-vector and sequenced to make sure to get the target sequence. Then, the large DNA segment was amplified, purified, and ligated into the pOK12 vector (Novagene, USA) between the *Kpn*I and *Xho*I DNA restriction enzyme sites to get the recombinant vector pOK-NLuc-DsRed using T4 DNA ligase (Thermo Scientific, USA).

### Transfection, Virus Rescue, and Plaque Purification

The genomic DNA of PRVTJ was extracted using the phenol–chloroform extraction method. Vero cells seeded in six-well culture plates were co-transfected with 1-μg pOK-DsRed-NLuc plasmid and 1-μg genomic DNA of PRVTJ strain using 4 μl of the X-tremeGENE HP DNA transfection reagent (Roche, USA) according to the manufacturer's instructions. The first generation recombinant viruses were collected at 2–3 days after transfection.

For plaque purification, PK-15 cells seeded in six-well cell culture plates were infected with 10-fold serially diluted rPRVTJ-NLuc strain from 10^−1^ to 10^−5^ for 1 h at 37°C in a 5% CO_2_ incubator; then, the supernatant was removed; cells were covered with 1% agarose gel and incubated for 2 days till clear cytopathic effect with red fluorescent of DsRed protein formed. Marked plaques were picked by pushing the 200-μl tip through the overlay agarose, and this was diluted in 1-ml DMEM for the next generation of plaque purification. A total of five generations of plaque purification were performed to obtain the purified recombinant viruses.

### Virus Titration and One-Step Growth Assay

The viral titer was determined by 50% cell culture infectious dose (TCID_50_). In brief, PK-15 cells seeded in 96-well plates were infected with 10-fold serially diluted viruses (10^−2^ to 10^−8^) and cultured at 37°C in a 5% CO_2_ incubator for 72 h. The number of wells with red fluorescence was counted, and the viral titers were calculated using the Reed & Muench method (21).

One-step growth kinetic of rPRVTJ-NLuc was compared with PRVTJ. The monolayers of PK-15 cells in the 24-well cell culture plates were infected with rPRVTJ-NLuc or PRVTJ at a multiplicity of infection (MOI) of 10 for 1 h at 37°C. Extracellular viruses were inactivated by low-PH treatment (22); supernatant and cells were harvested at different time points (12-h interval) till 60-h post-infection; three repeat samples were harvested at each time point and stored at −80°C. After the sample returned to room temperate, the cellular debris was removed by centrifugation, and the TCID_50_ of supernatant was detected on PK-15 cells. Average values and standard deviations of the three independent experiments were calculated.

### Polymerase Chain Reaction

Genomic DNA was extracted from PK-15 cells infected with rPRVTJ-NLuc or PRVTJ at an MOI of 1 for 12 h using the Tissue DNA Kit (Omega). The primer pairs P5S/P5R, P6S/P6R, and P7S/P7R that were complementary to the glycoprotein B (gB), glyco-protein I (gI), NLuc luciferase, and DsRed fluorescent protein genes are listed in Table 1. The amplification was conducted in a total volume of 50 μl containing 25 μl of 2× PrimeSTAR buffer (TaKaRa, Japan), 3 μl of DNA, 9.5 μl of sterilized water, and 1.0 mM for each primer. The reaction was heated at 95°C for 5 min, followed by 35 cycles of 98°C for 10 s, 58°C for 30 s, and 72°C for 2 min, with a final elongation step of 72°C for 10 min. The PCR product was analyzed using 1.0% agarose gel electrophoresis.

**Table 1:** Sequences of oligonucleotides used in PCR.

**Fragments**	**Primers**	**Sequences of primers (5^′^-3^′^)**	**Length (bp)**
L arm	P1S	GGTGCCTGCTGTACTACGTGTACGAGCCCTGCATC	1274
	P1R	CTACACGTGCCTGGCGACGATGCC	
R arm	P2S	CGAGCGAGCGAGCGAACGGGAG	1023
	P2R	CTAGGAGATGGTACATCGCGGGGCGCGCTCGCG	
CMV-DsRed-polyA	P3S	TAGATAACTGATCATAATCAGCCATACCA	1486
	P3R	CGCCGTTTAAACGCAGTGAAAAAAATGCTTTA	
NLuc	P4S	GTCTTCACACTCGAAGATTTC	513
	P4R	TTACGCCAGAATGCGTTCGCAC	
gB	P5S	GGGGTTGGACAGGAAGGACACCA	198
	P5R	AACCAGCTGCACGCGCTCAA	
gI	P6S	TGGCTCTGCGTGCTGTGCTC	343
	P6R	CATTCGTCACTTCCGGTTTC	
DsRed	P7S	ATGGCCTCCTCCGAGAACG	747
	P7R	TTATCTAGATCCGGTGGAACCCG	

### Western Blot Assay

The monolayers of PK-15 cells in the 6-well cell culture plates were infected with rPRVTJ-NLuc, rPRVTJ-DsRed, or PRVTJ A at an MOI of 1 at 37°C for 24 h. The total protein of cells was collected after adding an NP40 lysis buffer containing 1% phenylmethylsulfonyl fluoride (Solarbio, Beijing, China) prot, separated by sodium dodecyl sulfate-polyacrylamide gel electrophoresis, and transferred onto nitrocellulose membranes. The membranes were blocked with 5% skim milk for 2 h at 37°C and incubated at room temperature for 2 h with specific mouse anti-gD, an anti-gB monoclonal antibody (a gift of Jing Zhao, State Key Laboratory of Veterinary Biotechnology, Harbin Veterinary Research Institute, Chinese Academy of Agricultural Sciences, Harbin, China) and anti-DsRed polyclonal antibody (Solarbio, Beijing, China). The membranes were washed with phosphate-buffered saline (PBS) with Tween buffer for three times and incubated with DyLight 800 goat anti-mouse IgG (1:8,000) (Thermo Fisher Scientific) at 37°C for 45 min; the membranes were washed for another three times, then visualized and analyzed with an Odyssey infrared imaging system (LI-COR Biosciences, Lincoln, NE, USA).

### Luciferase Assay

PK-15 cells seeded in 96-well cell culture plates were infected with 10-fold serially diluted rPRVTJ-NLuc strain from 10^0^ to 10^−5^ at 37°C in a 5% CO_2_ incubator for 12 h. The supernatants were removed, and cells were washed once with PBS before cell culture lysis buffer (Promega) was added. Cell lysates were assayed for luminescence activity with the Nano-Glo Assay System (Promega), and luminescence was detected with TD-20/20 luminometer (Turner Designs).

### Infection of Mice With rPRVTJ-NLuc or PRVTJ and Tissue Collection

All the mice were handled according to the Guide for the Care and Use of Laboratory Animals of Harbin Veterinary Research Institute (HVRI), Chinese Academy of Agricultural Sciences, China. Two mice experiments were made in this work: one for the pathogen detection and another one for the live imaging test. For the pathogen detection, 35 6-week-old specific-pathogen-free (SPF) female BALB/c mice were used in this study. The mice were randomly allocated into seven groups, five mice in one box. Mice of groups 1, 2, and 3 were injected intramuscularly (i.m.) with 10^4^, 10^3^, or 10^2^ TCID_50_ of PRV TJ strain in 100-μl DMEM, respectively. Groups 4, 5, and 6 were injected i.m. with 10^4^, 10^3^, or 10^2^ TCID_50_ rPRVTJ-NLuc in 100 μl of DMEM, respectively. Group 7 was the mock-inoculations (medium only) in parallel. Mice were scored daily for symptoms of PRV infection using the following three-point system adapted from the protocol previously described (23). All the mice in the infected and control groups were anesthetized by CO_2_ before euthanasia, using the broken-neck method at 7 dpi. Fresh tissues from the heart, liver, spleen, lungs, kidneys, brain, spinal cord, and trigeminal ganglion of mice were collected, one part of the samples was fixed in buffered formalin for hematoxylin and eosin assay, and 100 mg of another part of the samples was stored at −80°C for DNA extraction. Total DNA was extracted using Tissue DNA Kit (Omega) according to the manufacturer's instructions and stored at −20°C for quantitative PCR (qPCR) analysis.

### Real-Time PCR (Quantitative PCR)

Real-time PCR (qPCR) was used to quantitatively analyze the viral loading in the brain, heart, liver, spleen, lung, kidney, spinal cord, and trigeminal ganglion using the reported method of Meng (24). Briefly, viral genomic DNA was amplified using the *gI* gene-specific primer PRV-F1 (5′-GCC GAG TAC CTC TGC C-3′), PRV-R1 (5′-CGA GAC GAA CAG CCG-3′), and TaqMan probes HEX-PRV-Var (HEX-5′-CCG CGT GCA CCA CGA AGC CT-3′-BHQ1); each sample was done in triplicate, deionized water as the negative control.

### Pathology and Histopathology

The samples were fixed in buffered formalin and embedded in paraffin wax. Tissue sections were prepared and stained with hematoxylin and eosin assay for histopathological examinations.

### *In vivo* Imaging

For the live imaging test, a total of 30 7-week-old SPF female BALB/c mice were separated into six groups for PRV infection, five mice in each group, and another five mice were injected with DMEM as control. Mice were infected with 10^3^ TCID_50_ rPRVTJ-NLuc or PRVTJ by three different inoculation routes: inoculated intraperitoneally (i.p.) (in the lower abdominal region), i.m. (in the right hind leg muscles), or subcutaneously (s.c.) (in the back of the neck). *In vivo* imaging was performed from 3 to 7 dpi with a 24-h interval using the BLI system of the LB 983 NightOWL II (Berthold, Germany) equipped with a cooled slow-scan CCD camera and driven by the IndiGo^TM^ software (version 2.0.5.0, Berthold). At each time point, five mice in each group per day were anesthetized with isoflurane (Burbank, CA 91502, USA) and injected with 100 μl of Nano-Glo reagent (Promega) (diluted 1:20 in PBS) *via* the tail vein. Flux measurements were acquired from regions of interest automatically gated to the signal contours, keeping the mice at 37°C during the whole progress. All the mice in the infected and control groups were anesthetized by CO_2_ before euthanasia, using the broken-neck method after imaging the experiment. All data in composite images utilized the same scale.

### Statistics

Data represent means ± standard deviations (*n* ≥ 3). The comparison between groups was performed by a Student's *t*-test with a two-tailed analysis. Data are considered significant when *P* < 0.05.

## Results

### Generation and Characterization of the Recombinant Pseudorabies Virus Expressing NanoLuc

**Figure 1:**
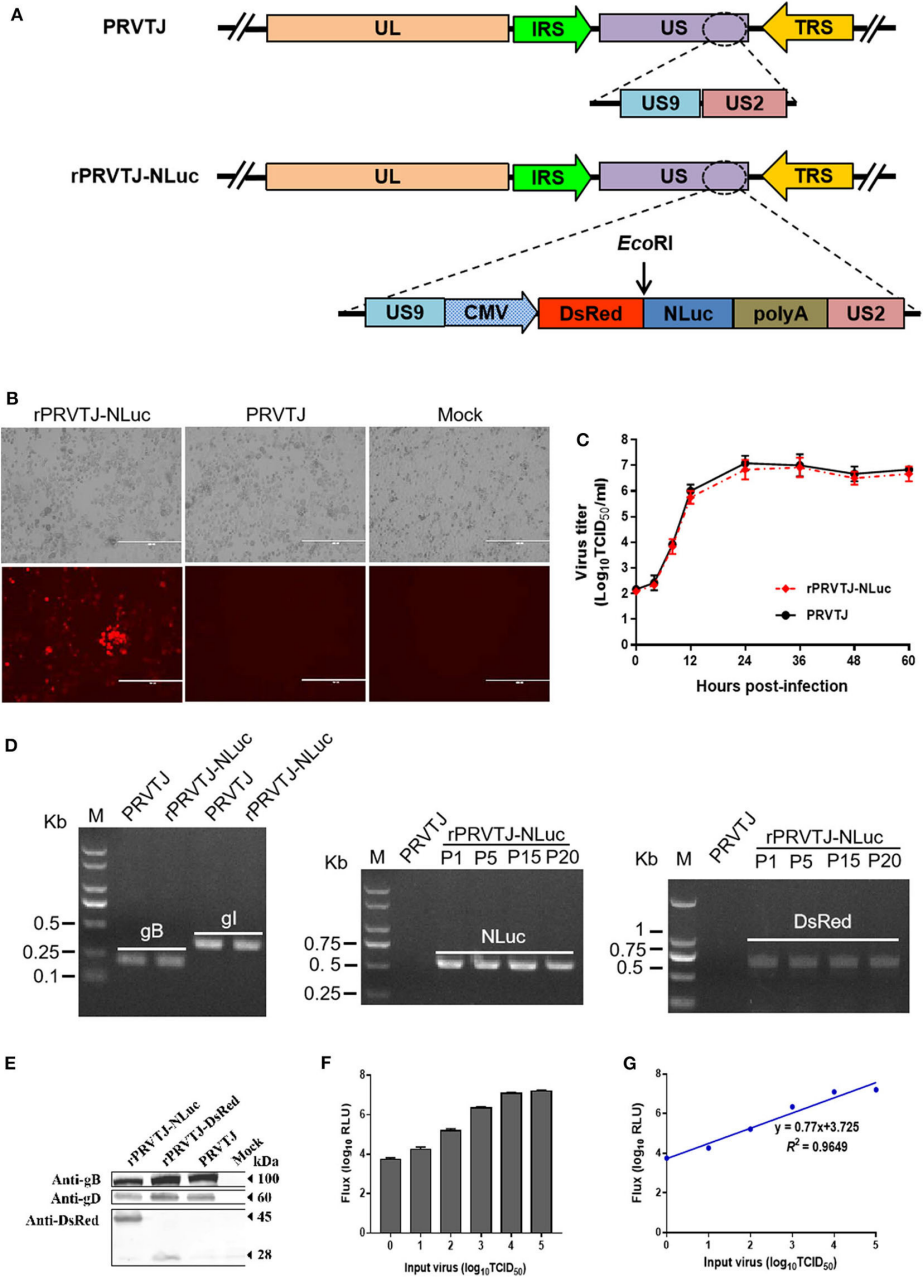
Generation and characterization of the recombinant PRV expressing NLuc luciferase. **(A)** Schematic representation of NLuc and DsRed cassette inserting immediately downstream of the *US9* gene. **(B)** Recombinant PRV can form plaques and express DsRed in the PRV-infected PK-15 cells. Original magnification ×200; bar, 400 μm. **(C)** One-step growth curve of rPRVTJ-NLuc. **(D)** Identify of rPRVTJ-NLuc by PCR. Left panel, verification of the *gB, gI* gene in the genome of rPRVTJ-NLuc, and PRVTJ. Middle panel and right panel, identification of the NLuc and DsRed reporter genes in different passages in infected PK-15 cells, respectively. **(E)** Expression of NLuc fused with DsRed in rPRVTJ-NLuc-infected cells was detected by Western blot, the DsRed expression of rPRVTJ-DsRed as control; moreover, viral protein gD and gB of all PRV strains were also detected using anti-gD and anti-gB monoclonal antibodies, normal cells as mock. **(F)** Average luciferase activity of the recombinant PRV in the PK-15 cells at 12 hpi with defined amounts of input virus (*n* = 5). **(G)** Correlation curve between titers of virus and luciferase activities using Pearson's correlation coefficient.

PRV genome is characterized by two unique regions (U_L_ and U_S_), and the U_S_ region flanked by internal and terminal repeat sequences. As reported, the noncoding interval sequence between U_S_9 open reading frame and U_S_2 open reading frame in the U_S_ regions is long enough to tolerate large exogenous genes (25). A single cassette of NLuc fused with the DsRed was designed immediately downstream of the *US9* gene of the PRV TJ genome (Figure 1A). The transfer vector pOK-DsRed-NLuc with two 1.5-kb homologous arms and PRVTJ genomic DNA were co-transfected into Vero cells, and the first generation of recombinant virus was collected at 2–3 days after transfection when the cytopathic effect formed on the Vero cells. We obtained the purified virus through five rounds of plaque purification. The plaque morphology of rPRVTJ-NLuc had no significant difference from its parental virus (Figure 1B). We confirmed that the chimeric NLuc cassette was inserted immediately into the downstream of the *US9* gene of PRVTJ using corresponding specific primers by PCR and sequencing. The stability of the reporter genes, NLuc, and DsRed, was also tested by following amplification and serial passages in PK-15 cells (P1 to P20) (Figure 1D). These results indicated there were *gB* and *gI* genes in the genome backbone of the recombinant PRV, which was the same as its parental strain. Also, the reporter genes could exist in the backbone simultaneously and stably, which were detected in the following passages. The expression of DsRed and viral protein was checked by Western blot using antibody target DsRed, gD, and gB proteins. The same bands of gB and gD presented in the samples of rPRVTJ-NLuc, rPRVTJ-DsRed, and PRVTJ infected groups; meanwhile, the band of DsRed protein of rPRVTJ-NLuc was larger than rPRVTJ-DsRed due to NLuc fused with it (Figure 1E).

We compared the growth kinetics of rPRVTJ-NLuc with the parental strain PRVTJ in PK-15 cells. The titers of rPRVTJ-NLuc in the culture supernatant had no significant difference with those of PRVTJ during 60-h post-infection (hpi), suggesting that the growth properties of rPRVTJ-NLuc were similar to those of PRVTJ (Figure 1C). To examine the relationship between NLuc expression level and rPRVTJ-NLuc infection, the correlation between the input of rPRVTJ-NLuc and NLuc luciferase activities was evaluated. PK-15 cells were infected with rPRVTJ-NLuc with different amounts of input virus: ranging from 10^0^ to 10^5^ TCID_50_/ml. NLuc luciferase activities were measured at 12 hpi. The activities of luciferase in supernatants increased over time, and the yield of luminescent flux was directly correlated with viral titers (*R*^2^ = 0.96) (Figures 1F,G). The result demonstrated that the luminescent flux could represent the titer of the recombinant PRV in the infected cell.

### rPRVTJ-NLuc Possesses Similar Pathogenicity to PRVTJ in Mice

**Figure 2:**
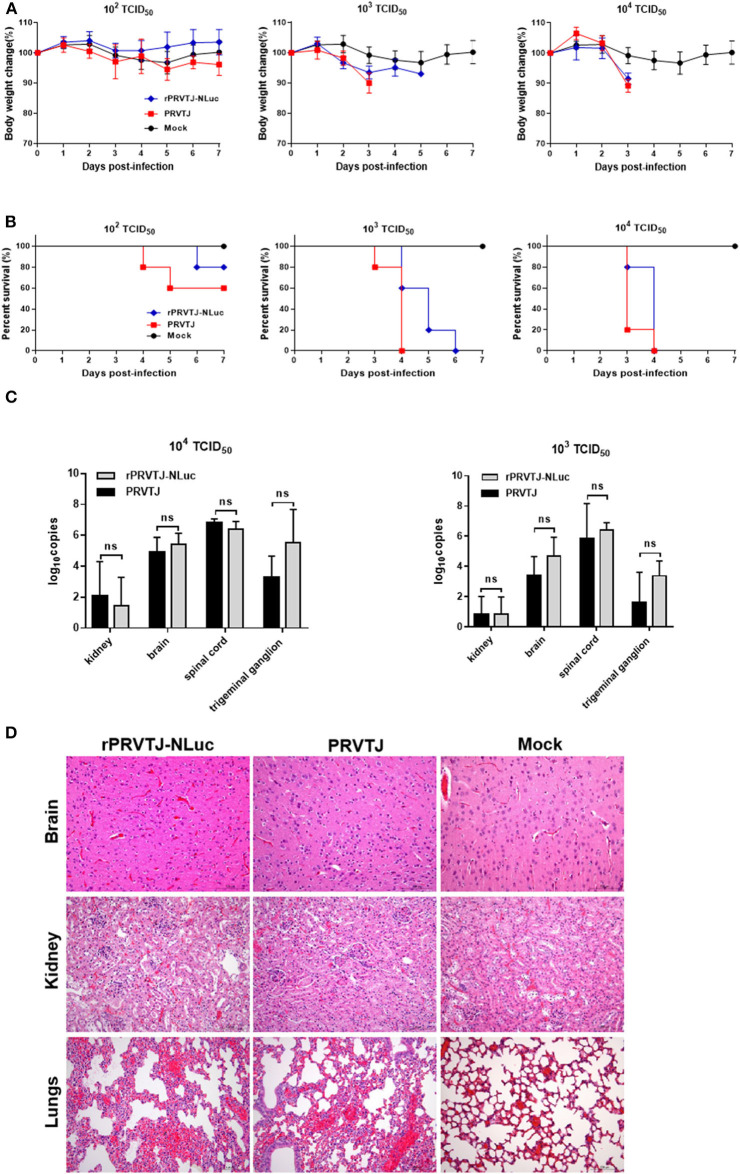
Pathogenicity of recombinant PRV compared with PRVTJ in mice. Six-week-old SPF BALB/c mice were injected intramuscularly with 10^4^, 10^3^, or 10^2^ TCID_50_ of rPRVTJ-NLuc or PRVTJ. **(A)** Average body weights of the infected mice (*n* = 5) at 7 dpi. **(B)** Survival rates of mice infected indicated a dose of viruses. **(C)** Viral DNA copies in different tissue. All the mock-infected and PRV-infected mice were euthanized and subjected to dissection at a moribund stage or 7 dpi. Specific tissues were collected from the mice infected with 10^4^ or 10^3^ TCID_50_ rPRVTJ-NLuc or PRVTJ and tested by qPCR. **(D)** Histopathological changes in diverse organs of mice infected with rPRVTJ-NLuc or PRVTJ; bar = 100 μm.

To monitor symptoms and organ lesions preferably, mice were infected with 10^4^, 10^3^, or 10^2^ TCID_50_ PRVTJ or rPRVTJ-NLuc *via* the i.m. route. Clinical signs, including pruritus, anxiety, rolling, and scratching of the injection site, were recorded every day throughout the experiment. The mice infected with 10^4^ TCID_50_ viruses (groups 1 and 4) began showing clinical signs around 72 hpi. The mice infected with 10^3^ TCID_50_ viruses (groups 2 and 5) showed similar symptoms but delayed. The mice infected with 10^2^ TCID_50_ doses of viruses (groups 3 and 6) showed barely clinical symptoms. No difference was observed in the clinical performance of mice injected with the same dose of PRVTJ and rPRVTJ-NLuc (Table 2). Mice infected with 10^4^, 10^3^, or 10^2^ TCID_50_ of PRVTJ or rPRVTJ-NLuc lost body weight and led to die in a dose-dependent manner (Figures 2A, B). The mice infected with the same dose of PRVTJ or rPRVTJ-NLuc exhibited similar weight loss and mortality. These results demonstrated that the clinical symptoms of the recombinant PRV did not change after the insertion of the reporter genes.

**Table 2:** Clinical signs score of the mice infected with rPRVTJ-NLuc or PRVTJ.

**Virus**	**Dose (TCID_50_)**	**Days post-infection**						
		**1**	**2**	**3**	**4**	**5**	**6**	**7**
PRVTJ	10^4^	0	0	11 ± 1	-	-	-	-
	10^3^	0	0	6.6 ± 0.55	-	-	-	-
	10^2^	0	0	4 ± 0.71	7.25 ± 0.5	4 ± 1	2 ± 1	2 ± 0
rPRVTJ-NLuc	10^4^	0	0	9.75 ± 0.96	-	-	-	-
	10^3^	0	0	4.5 ± 1	10 ± 1.73	5 ± 0	-	-
	10^2^	0	0	3 ± 0.71	5.8 ± 0.84	2.6 ± 0.89	2 ± 1	3 ± 0
DMEM	100 μl	0	0	0	0	0	0	0

*Extended scoring systems for clinical signs. Clinical signs were recorded every day, incorporating 4 parameters such as:pruritus, anxiety, rolling and scratching of the injection site, each parameter was scored from 0 = no clinical symptom; 1 = mild symptoms, such as subtle neurological symptoms, untidy hair and minor depression; 2 = common symptoms, such as pruritus, rolling and scratching the injection site; 3 = severe symptoms, such as severe pruritus, self-mutilate even acute death. The results are expressed as mean value ± s.d. (n = 5) of total value of 4 parameters. -, death*.

Next, we determined whether the fatal clinical outcome and distinct pathology of the mice that were infected could be attributed to viral replication in specific tissues. Various tissues, as listed earlier, were collected after euthanasia for qPCR analysis to determine PRV DNA loads. As shown in Figure 2C, the virus could be detected in the brain, kidney, spinal cord, and trigeminal ganglion of peracute death mice infected with 10^4^ or 10^3^ TCID_50_ PRVs groups (groups 1, 2, 4, and 5). No virus was detected in the 10^2^ TCID_50_ groups (groups 3 and 6), as well as the control group (group 7). PRV replication was detected principally in kidneys, brain, spinal cord, and trigeminal ganglion tissues after i.m. injection, but there was no significant difference in PRV DNA loads between rPRVTJ-NLuc and PRVTJ infected groups, which was consistent with the clinical signs score and pathological analysis, indicating that the replication and spread properties of rPRVTJ-NLuc were similar to those of the parental strain.

Histopathological examination of several tissues (brain, lungs, and kidneys) was performed to check the difference between the mice infected with PRVTJ and rPRVTJ-NLuc (Figure 2D), and tissue samples were taken from three mice in each group at the humane endpoint. Firstly, there were local hemorrhage, degeneration, necrosis of partial neuron, Purkinje cell, and glial cell proliferation in the brain of both PRV infected groups. The lungs of the PRV-infected groups showed congestion, the proliferation of alveolar epithelial cells, and a few lymphocytes infiltration. There were degeneration and necrosis of renal tubular epithelial cells in the kidneys of the PRV infected groups. Secondly, it is noteworthy that low-dose PRV infection induces significant histopathological changes in the central nervous system (CNS) of mice as well. The histopathological changes of these mice organs infected with PRVTJ or rPRVTJ-NLuc did not differ on every dosage. Finally, the heart, liver, and spleen of all the PRV-infected mice and all the mice infected with 10^2^ TCID_50_ (groups 3 and 6) PRV had no histopathological changes. Collectively, these data indicated that the insertion of NLuc into the PRV genome did not influence histopathological changes of PRV-infected mice.

In summary, there is no difference in clinical symptoms, replication, spread, and histopathology between the mice infected with rPRVTJ-NLuc and its parental virus. So, the insertion of NLuc into the PRV genome was stable, and the recombinant rPRVTJ-NLuc could be used as a tool to study the pathogenicity of PRVTJ.

### *In vivo* Imaging of rPRVTJ-NLuc Showed the Viral Replication and Dissemination in Mice

**Figure 3:**
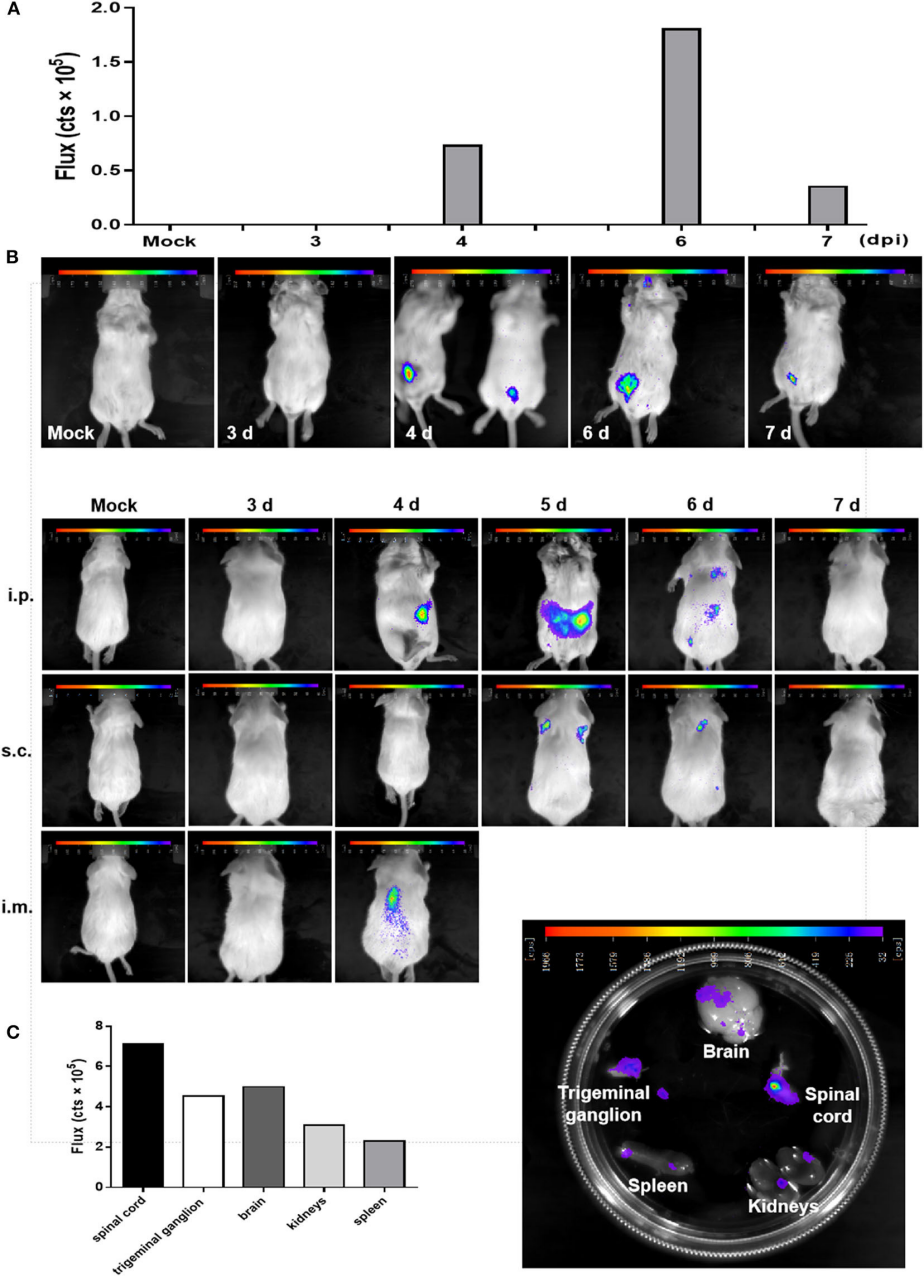
*In vivo* imaging of a PRV reporter in a mouse model. **(A)** Noninvasive imaging detected robust bioluminescence in the mice infected with 10^3^ TCID_50_ rPRVTJ-NLuc or PRVTJ and analyzed for bioluminescence at indicated time points. **(B)** Longitudinal *in vivo* imaging of rPRVTJ-NLuc in mice showed viral replication and dissemination. Mice were inoculated intraperitoneally, subcutaneously, or intramuscularly with 10^3^ TCID_50_ rPRVTJ-NLuc and PRVTJ. *In vivo* imaging was performed every day from 3 dpi at the indicated time points. **(C)** *In situ* localization of NLuc luciferase activity in the excised organs of mice infected with the recombinant PRV. Seven-week-old SPF BALB/c mice were i.p. inoculated with 10^3^ TCID_50_ rPRVTJ-NLuc and killed at 4 dpi. Organs were dissected out and placed separately in a substrate bath. Bioluminescent images were taken 10 min later, and the representative image shows the distribution of luminescent activity in the specimen (right). Quantification of the luminescent activity in the excised organs. After an exposure time of 30 s, a region of interest was manually defined, and the luminescent activity was calculated by the IndiGo^TM^ software (left). All the images of the mice shown in the figure were one representation of five repeats.

BLI has been exploited in multiple viruses, including dengue virus, herpes simplex virus type 1, Sindbis virus, and Sendai virus (15, 17, 18). Here, we used rPRVTJ-NLuc to visualize PRV replication and spread in mice. Seven-week-old BALB/c mice were infected with 10^3^ TCID_50_ rPRVTJ-NLuc and subjected to daily imaging (Figure 3A). Bioluminescence was detected as early as 4 dpi at the site of injection. Luminescent flux increased throughout infection and peaked on day 6, then waning. The enhanced signal could be detected when rPRVTJ-NLuc spread from the injection site to the superior respiratory tract.

To get better *in vivo* images, mice were infected with rPRVTJ-NLuc and PRVTJ by three different inoculation routes. Mice were infected with 10^3^ TCID_50_ viruses *via* inoculated i.p. (in the lower abdominal region), i.m. (in the right hind leg muscles), or s.c. (in the back of the neck). As expected, mice infected with rPRVTJ-NLuc displayed robust bioluminescence, which started 4 dpi, increased over time, peaked at 5 dpi, remained detectable for at least 6 dpi (Figure 3B), and disappeared at 7 dpi when the virus was cleared after a sublethal infection. All mice inoculated viruses *via* i.m. route showed severe clinical symptoms at 4 dpi, pruritus, rolling, and scratching the injection site, and all died at 5 dpi. This result illustrated that the i.m. route may more easily cause peracute death. Real-time imaging of the same mouse over time from each group revealed viral load and dissemination from the injection site to the spinal cord and CNS.

To confirm the local tissues scattering bioluminescence, selected mice were killed, and their organs were immediately removed. Isolated organs were placed in a furimazine bath, and BLI was undertaken. Signals of the spinal cord, trigeminal ganglion, and a partial region of the brain were observed (Figure 3C). There were robust bioluminescence signals in the spinal cord, brain, and trigeminal ganglion based on the flux of each excised organs (Figure 3C). These results were consistent with the viral distribution in diverse organs of mice infected with rPRVTJ-NLuc, which possessed the ability to visualize a pathogenic infection in mice.

## Discussion

Since late 2011, there were PR-like outbreaks in a large number of vaccinated pig farms in China (26). It is found that the PRV variant strain and the classical strain belong to different phylogenetic branches. In our previous study, the epidemic strain exhibited enhanced pathogenicity in mice and pigs (6). The reemergence of the PRV outbreak resulted in huge economic losses to the pig industry in China, so the ability that can quickly assess the pathogenicity of the epidemic strains and its susceptibility to antiviral interventions was urgently required.

In this study, we constructed a recombinant PRV stably expressing the NLuc luciferase fusion with red fluorescent protein DsRed. The double-labeled strategy is convenient for *in vitro* screening of the recombinant virus with DsRed and the *in vivo* imaging with NLuc luciferase. There was no difference in the growth properties of rPRVTJ-NLuc and PRVTJ. Moreover, the recombinant rPRVTJ-NLuc possessed pathogenicity and lethality in mice indistinguishable from those of PRVTJ.

The infection of PRV in rodents routinely shows severe clinical manifestations, including pretty itchy, frantically clawing, and biting of the inoculation site, even self-mutilation and acute death eventually (27). To monitor the symptoms and organ lesions preferably, we infected mice with a lower dosage, 10^4^, 10^3^, or 10^2^ TCID_50_ PRVTJ, or rPRVTJ-NLuc *via* i.m. route. Similar symptoms were found in mice infected with 10^4^ or 10^3^ TCID_50_ PRVTJ or rPRVTJ-NLuc; the groups of 10^2^ TCID_50_ viruses and control were no symptomatic; all these results were the same as Brittle reported. Previous studies demonstrated that mice infected with PRV-Becker develop severe pruritus in the inoculation site, resulting in self-mutilation and peracute death with no detectable behavioral CNS pathology and no obvious pathological changes in the brain. Merely, the peripheral nervous system (dorsal root ganglia and trigeminal ganglia) has significant pathological changes (2, 28). Nevertheless, we detected the replication of the virus and inflammatory pathological changes in CNS tissue and kidneys of mice; maybe, these explained why PRVTJ has higher pathogenicity than the classic PRV strains.

The sensitivity and noninvasive longitudinal measurements enable the monitoring of PRV infection and clearance of a lower-dose infection of rPRVTJ-NLuc in mice (Figures 3A,B). Actually, 6-week-old mice were tended to death because of PRV infection. To obtain continuously spatial and temporal progression of PRV infection, the 7-week-old mice were used to perform *in vivo* imaging. In a mouse inoculated i.p. with the dose of 10^3^ TCID_50_, the infection began primarily in i.p. injection site. By 6 dpi, the bioluminescence signal decreased and appeared in the position of the spinal cord, indicating the spread of the virus to the CNS. We confirmed the infection of the spinal cord by imaging of excised organs and qPCR (Figure 3C), showing stronger bioluminescence and higher virus load in the spinal cord than *in vivo* image. The infection continued to be cleared and was undetectable at 7 dpi. This longitudinal measurement course could detect the dynamics of PRV by *in vivo* imaging and elucidate mechanisms of viral dissemination in a mouse model. It is compelling that a series of reporter virus expressing the NLuc luciferase has the potential to assess the pathogenicity and properties of viral replication and *in vivo* dissemination of diverse PRV stains. Thus, this strategy should be widely applicable to any PRV isolate and will be useful to rapidly access the replication and pathogenicity characteristics of emerging PRV strains.

Although the BLI potentially offers significant advantages over other traditional virological methods, it also has a few disadvantages, e.g., the attenuation of the signal by hair and organ pigmentation, overlapping signals, and the attenuation of signals due to organ depth from the surface. Bioluminescence was not detected in the brain in any of our experiments of whole-body imaging, which is most probably due to the signal attenuation caused by the skull of mice. We confirmed the bioluminescence signal in the brain and trigeminal ganglion by imaging of excised organs. The result is consistent with detectable viral distribution in diverse tissues. Therefore, these data show that although bioluminescence is promising, precise viral tracking and the exact quantification of viral titers require combinations of these methodologies.

In summary, the recombinant PRV stably expressing NLuc luciferase is shown to be a potential tool for studying the pathogenicity mechanism of the PRV variant. We further showed that the recombinant PRV could be applied to study the infection mechanisms of PRVTJ strain by BLI. In further studies, the reporter virus will be used in the research of pathogenicity mechanism diversity of PRV variants and classic strains.

## Data Availability Statement

All datasets generated for this study are included in the article.

## Ethics Statement

All the animal experiments were approved by the Committee on the Ethics of Animal Experiments of the State Key Laboratory of Veterinary Biotechnology, Harbin Veterinary Research Institute, Chinese Academy of Agricultural Sciences. Five SPF BALB/c mice were breed in one box, mice were euthanized using sodium pentobarbital anesthetic before living-image and all recommended efforts were taken to minimize suffering.

## Author Contributions

YS and H-JQ designed research. YW and HW performed experiments. BW wrote the manuscript. The authors YW, HW, and BW have the equal contribution for this work and all authors reviewed the manuscript. All authors contributed to the article and approved the submitted version.

## Conflict of Interest

The authors declare that the research was conducted in the absence of any commercial or financial relationships that could be construed as a potential conflict of interest.
